# Compression or expansion of dementia in Germany? An observational study of short-term trends in incidence and death rates of dementia between 2006/07 and 2009/10 based on German health insurance data

**DOI:** 10.1186/s13195-015-0146-x

**Published:** 2015-11-05

**Authors:** Gabriele Doblhammer, Anne Fink, Stephanie Zylla, Frans Willekens

**Affiliations:** University of Rostock, Institute for Sociology and Demography, Ulmenstr. 69, 18057 Rostock, Germany; German Center for Neurodegenerative Diseases (DZNE) Bonn, Ludwig-Erhard-Allee, 53175 Bonn, Germany; Max Planck Institute for Demographic Research, Konrad-Zuse-Str. 1, 18057 Rostock, Germany; Rostock Center for the Study of Demographic Change, Konrad-Zuse-Str. 1, 18057 Rostock, Germany

## Abstract

**Introduction:**

There have been recent reports about a decline in dementia incidence, but only little is known about trends in the mortality of patients with dementia. Only the simultaneous analysis of both trends can inform whether the reported decline in dementia has led to a compression of dementia into higher ages.

**Methods:**

We used health claims data from the largest public health insurer in Germany over the two time periods 2004/07 and 2007/10. Dementia was defined according to the International Classification of Disease 10th revision (ICD-10) numbers G30, G31.0, G31.82, G23.1, F00, F01, F02, F03 and F05.1 or by a prescription of cholinesterase inhibitors or memantine or both. In the two time periods, we observed 502,065 person-years of exposure and 10,881 incident dementia cases and 10,013 person-years of exposure among the newly demented and 3049 deaths. We estimated the relative risks of the two time periods applying proportional hazard models and calculated years with or without dementia using the illness-death model.

**Results:**

Dementia incidence was significantly higher in 2006/07 than in 2009/10, whereas mortality with dementia tended to be lower in the first period, albeit statistically significant among women only. Mortality without dementia tended to be higher in the first period for men and remained stable for women. Combining these trends, we found that at age 65 remaining life years with dementia were compressed by a yearly 0.4 months for men and 1.4 months for women. At the same time, remaining life years without dementia increased by a yearly 1.4 months for men and 1.1 months for women.

**Conclusions:**

This study provides evidence that the increase in dementia-free life years went together with an absolute compression of life years with dementia. This positive trend was particularly strong among women. Results were controlled for trends in multi-morbidity and care need, suggesting that the postponement in dementia incidence is not simply caused by a delay in diagnosis.

**Electronic supplementary material:**

The online version of this article (doi:10.1186/s13195-015-0146-x) contains supplementary material, which is available to authorized users.

## Introduction

The number of dementia cases worldwide is expected to double every 20 years, resulting in 65.7 million individuals with dementia by 2030 and 115.4 million by 2050 [1]. With 1.4 million patients with dementia in 2012, Germany belongs to the top 10 countries with the largest number of patients with dementia worldwide. The number is forecasted to increase up to three million by 2050 [2, 3]. A recent study showed that a yearly 1 % decline in dementia prevalence can counterbalance the effect of life expectancy (LE) increase on the number of patients with dementia, resulting in considerably less additional dementia cases than previously projected [2].

This interplay between incidence of dementia and mortality of patients with dementia is of major interest in terms of the broader framework of compression versus expansion of morbidity. Any extra years of life from an overall increase in LE could be spent in good health, which is referred to as compression of morbidity [4], or in poor health termed expansion of morbidity [5]. Alternately, unhealthy years may increase but do so as the proportion of life spent healthily is increasing/decreasing, resulting in a relative compression/relative expansion [6]. Finally, morbidity might increase at a rate similar to LE but severity might not, which is referred to as “dynamic equilibrium” [7].

Recent secular trends in dementia suggest a decrease in incidence rates, combined with stable or decreasing prevalence. Turning to prevalence studies, Manton et al. [8] observed a decline in severe cognitive impairment in the US between 1982 and 1999; Langa et al. [9] observed a continued decline between 1993 and 2002. There was improved cognitive functioning among Danish [10] and US [11] centenarians. Declining dementia was found by Matthews et al. [12] in three regions in the UK between 1981/94 and 2008/11 and by Doblhammer et al. [3] in Germany between 2007/09. In brain autopsies for the period 1972 to 2006, the average amyloid stage decreased in cases without clinically diagnosed dementia or cognitive disorders [13].

A number of recent incidence studies provided evidence of declining trends. Schrijvers et al. [14] showed a statistically not significant decline in incidence between 1990 and 2000 in Rotterdam, and Qiu et al. [15] inferred a decline in incidence between 1987/89 and 2001/02 from a stable prevalence combined with decreasing mortality in Stockholm.

Little is known about trends in the mortality of patients with dementia. Studies based on death certificates indicated increased mortality due to dementia and Alzheimer’s dementia in the 1980s and the 2000s [16, 17]. Langa et al. [9] compared the Health and Retirement Survey (HRS) of 1993–1995 with that of 2002–2004, finding a mortality increase among the highly educated for those with moderate or severe cognitive impairment. Using the HRS, Reuser and Willekens [18] showed that people with high levels of education have lower dementia incidence, but higher mortality. Qiu et al. [15] found increasing survival of patients with dementia in Stockholm.

In this study, we explored trends in the incidence of dementia and in the mortality with and without dementia. Combining this information, we estimated life years with and without dementia to explore the presence of a compression or expansion of dementia. We controlled for trends in co-morbidity and care need to test whether a shift in the general health status of the population may explain these trends. Based on the results of the prior studies, a decline in dementia incidence would be expected. Given past contradictory research results, no explicit hypothesis about the trend in mortality of patients with dementia was formulated. General improvements in health and survival may reduce the incidence of dementia [19] but may also benefit patients with dementia in terms of their co-morbidities, possibly increasing their LE [20]. On the other hand, increasing educational levels may lead to increasing cognitive reserve [21]. Cognitive reserve is a key concept in the study of cognitive functioning. It stems from the repeated observation that there is no straightforward relationship between the degree of brain pathology and cognitive functioning. A high level of educational attainment or a demanding occupation allows individuals to process tasks in a more efficient manner and thus to sustain greater brain damage before displaying major functional deficits. Increasing cognitive reserve would result in a later diagnosis [22] and in what would appear to be shorter survival rates.

We also examined the distribution of the new dementia cases. A decline in age-specific dementia incidence should go hand in hand with a postponement of dementia to higher ages, thus increasing the mean age of the new dementia cases.

## Methods

### Study design and participants

We compared two random samples of health claims data from Germany’s largest public health insurance, the “Allgemeine Ortskrankenkasse” (AOK), which covers about one third of the total population at least 50 years old and 50 % of the population at least 80 years old. An age-stratified 2.2 % random sample of the insured population at least 63 years old was drawn from all insured persons in the first quarter of 2004 and tracked through the fourth quarter of 2007. Incident dementia was defined for all individuals without a dementia diagnosis in the 2-year period 2004/05 and a first dementia diagnosis in 2006 or 2007. Thus, at the time of diagnosis, they would be at least 65 years old. A second independent 2.2 % random sample was drawn in the first quarter of 2007 with follow-up through the last quarter of 2010. Incident dementia was defined by a dementia-free period in 2007/08, and a first dementia diagnosis in 2009 or 2010. Only validated dementia cases were considered (for the validation procedure, see below). Death rates were estimated for the two time periods 2006/07 and 2009/10 for individuals with an incident dementia diagnosis only; separate death rates were estimated for those without dementia. The data included information about sex, age, all inpatient and outpatient diagnoses coded by International Classification of Disease 10th revision (ICD-10), prescriptions of medications filled on a quarterly basis, and whether patients received benefits or services from the German statutory long-term care (LTC) insurance.

Data access was legally approved by the “Wissenschaftliches Institut der Ortskrankenkassen” (WIdO). The study is based on anonymised administrative claims data that never involved patients directly. Individual patients cannot be identified, and the analyses presented do not affect patients whose anonymized records were used.

### Measurement of dementia

Dementia was defined by the ICD-10 numbers G30, G31.0, G31.82, G23.1, F00, F01, F02, F03, and F05.1 or by a prescription of cholinesterase inhibitors or memantine (or both), both of which are approved anti-dementia drugs. We did not distinguish according to aetiology, but combined all ICD codes into one group named “dementia”. To account for false-positive diagnoses of the true occurrence of dementia [23], we developed a validation procedure. First, only diagnoses indicated as “verified” by a medical doctor were included from outpatient services, whereas from inpatient services only the discharge and secondary diagnoses were considered. Second, only those diagnoses with a second occurrence in the same quarter by different types of physicians or over time were considered. The only exception was when a patient died immediately after a dementia diagnosis in the same quarter; all of these cases were considered valid dementia cases.

### Co-variates

We measured changes in the general health status of the population by controlling for the diagnosis of hypertension (ICD-10 Codes: I10-I15), diabetes mellitus (E10-E14), ischemic heart diseases (I20-I25), cerebrovascular diseases (I60-I69), hypercholesterolemia (E78), and atrial fibrillation (I48). We used benefits from the German public LTC insurance as an indirect measure of the severity of a dementia diagnosis. The insurance was established in 1995 and is financed as a pay-as-you-go system. To claim LTC benefits or services from the insurer, individuals have to make an application and pass an objective assessment, which is based mainly on impairments in activities of daily living. Applicants are assigned to one of three LTC levels: considerable, severe, or extreme. People who have a considerable level of LTC require care for at least 90 min per day, of which at least 46 min are needed for basic activities like washing, eating, or mobility. People with a severe level of LTC require care for at least 3 h per day, with at least 2 h needed for basic activities. People with an extreme level of LTC require care for at least 5 h per day, of which at least 4 h are needed for basic activities. LTC here comprises day care, home care by nurses or non-professionals as well as care in a nursing care home.

### Statistical analyses of trends in incidence and death rates

We estimated the relative risks (RRs) of dementia incidence and mortality with/without dementia in 2006/07 compared with 2009/10 by applying proportional hazard models with constant baseline hazards defined over the calendar time of the two time periods. The timescale of the baseline hazard is months since 2006 (2009). The models include an indicator variable for both time periods, age in 1-year age groups (defined as a second-degree polynomial and centered at age 80, the middle of the age interval), the co-morbidities, and the care level differentiated into none (care level 0), considerable (care level I), severe (care level II), and extreme (care level III). All covariates are specified as time-dependent variables. Individuals are censored at the time of their death (incidence model), their exit from the insurance company (incidence and mortality models), or the end of the observation period (incidence and mortality models), whichever occurred first. The proportionality assumption was tested by estimating the -ln(-ln) Kaplan-Meier survivor curves [24]. All proportional hazard models were estimated separately for both sexes. We conducted sensitivity analyses to test whether the results depended on the parametric form of the baseline hazard using a piecewise constant hazard rate, which was not the case. We also ran models using age at baseline applying Cox proportional hazard models which, again, did not alter the results. Whereas death rates were higher, the time trend remained unchanged. We used the procedure “streg” in STATA 12.1 for the estimation of the hazard models and the post-estimation command “predict” for the calculation of the hazard rates fitted by the models.

### Estimating life years with and without dementia

We used the illness-death model, which has been frequently applied to dementia research [20], to estimate life years with and without dementia and explore compression of dementia. The model is defined by two living states, non-demented and demented, and three period and sex-specific transitions: non-demented to demented, non-demented to death, and demented to death. We estimated the age-specific transition rates by fitting separate hazard models to the two time periods and the two sexes, only including the second-degree age-polynomials. We estimated the average LE at age 65 with dementia (LE _dementia 65_) and without dementia (LE _w/o dementia 65_) as well as the distribution of the new dementia cases *D* (*x*) at age x (for model details see “Additional file 1”). We bootstrapped 95 % confidence intervals by re-sampling the sample data with replacement. We performed a thousand replications each time estimating the age-specific transition rates by the hazard models and the LEs by the illness-death models.

## Results

### Time trends estimated with proportional hazard models

**Fig. 1:**
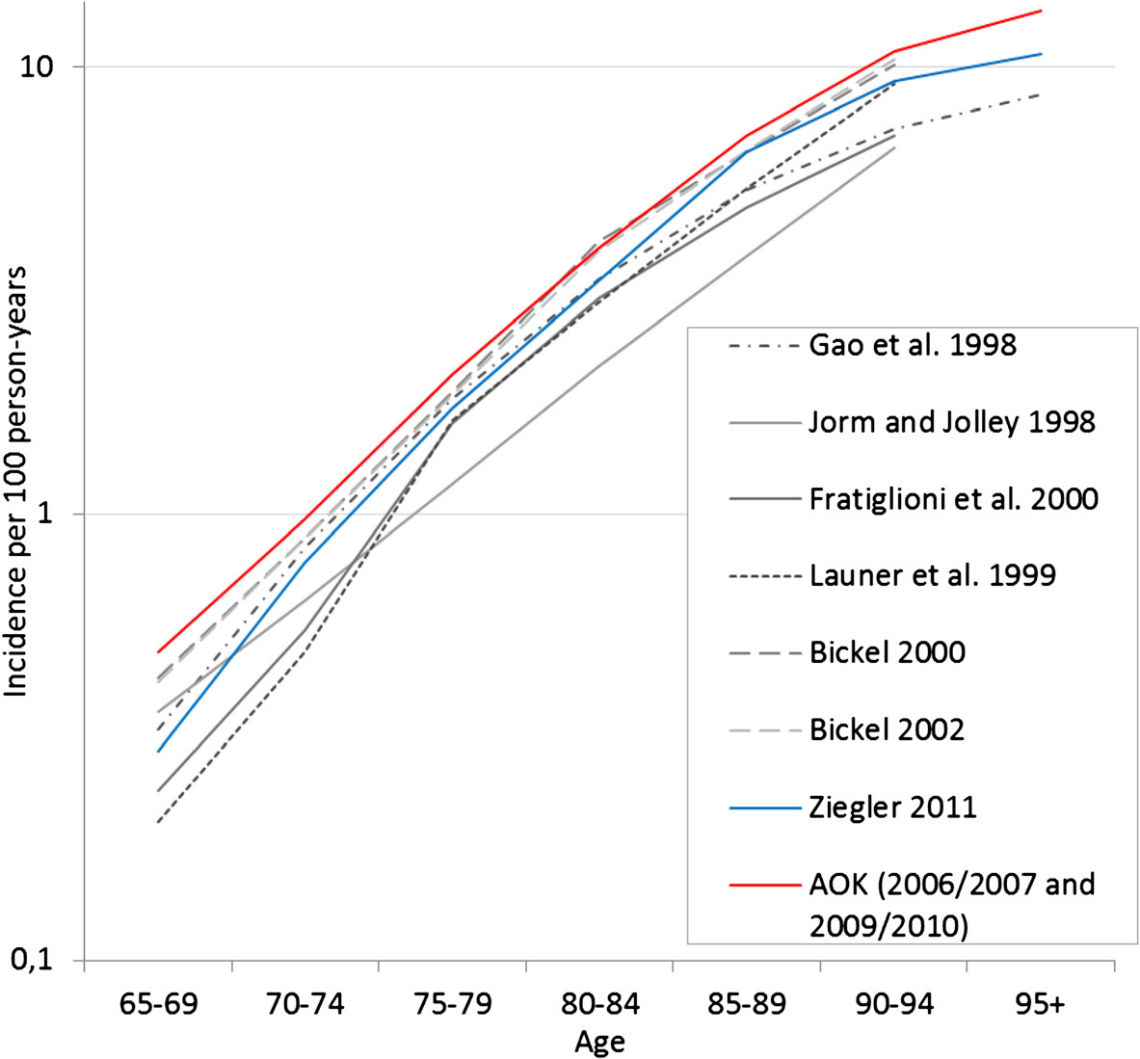
Comparison of dementia incidence rates (AOK 2006/07 and 2009/10) with previous studies. *AOK* Allgemeine Ortskrankenkassen

In the two time periods, there were a total of 10,881 incident dementia cases with 502,065 person-years of exposure (PYE). Among the non-demented, we observed 16,961 deaths with 500,714 PYE, among the newly demented 3049 deaths with 10,013 PYE (Table 1). Age-specific numbers and rates are given in the supplement (“Additional file 2: Table S1”). Total death rates were slightly higher as compared with the human mortality database [25], reflecting the bias toward lower social strata insured with the AOK. As expected, dementia incidence was slightly higher but highly consistent with results from previous studies (for an overview, see [26]) (Fig. 1).

**Table 1:** Descriptive statistics of the number of insured individuals, exposures in person-years and cases at ages 65 to 95+ in the two time periods by sex

Model		2006/2007		2009/2010		Total
		Men	Women	Men	Women	
Dementia incidence	N	56,011	85,081	54,257	80,986	276,335
	Exposures ^+^	100,034	154,962	98,358	148,711	502,065
	Incident dementia	1811	3768	1781	3521	10,881
Mortality without dementia diagnosis	N	55,684	84,377	53,955	80,357	274,373
	Exposures ^+^	99,806	154,498	98,136	148,274	500,714
	Deaths	3993	4463	4001	4504	16,961
Mortality with incident dementia diagnosis	N	1810	3769	1781	3520	10,880
	Exposures ^+^	1594	3694	1497	3228	10,013
	Deaths	579	912	598	960	3049

^+^in person years

**Fig. 2:**
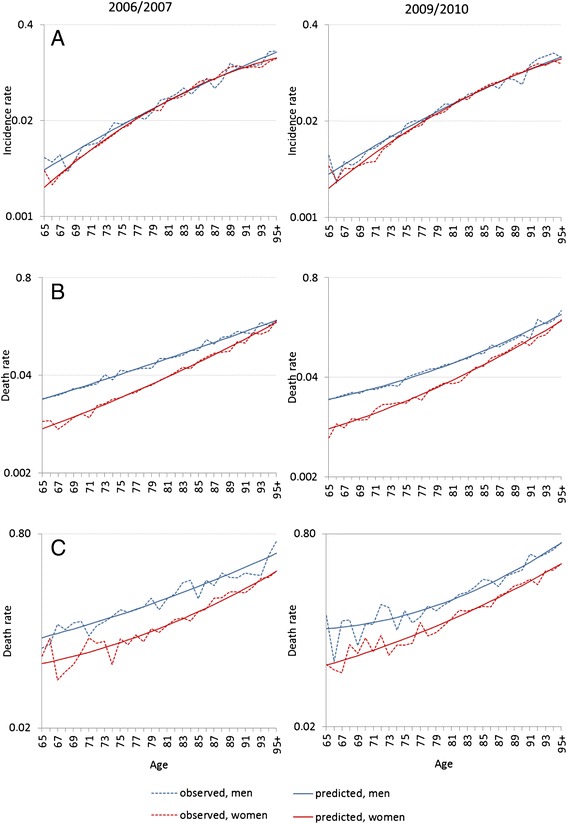
Observed and predicted values for (**a**) dementia incidence rates, (**b**) death rates of the demented, and (**c**) death rates of the non-demented for the periods 2006/07 and 2009/10 by sex (logarithmic scale)

The RRs of the two time periods estimated by proportional hazard models confirmed highly significant changes in the incidence of dementia as well as in death rates of demented women (Table 2). For both sexes, the incidence of dementia was significantly higher in the first period of 2006/07 (RR men: 1.10, *P* value = 0.006; RR women: 1.10, *P* value = 0.000) than in 2009/10. For men, mortality without a dementia diagnosis was somewhat higher in the first than the second period (RR men: 1.04, *P* value = 0.075), whereas mortality for women remained stable (RR women: 1.00, *P* value = 0.935). Mortality with a dementia increased for both sexes; in the first period, it was somewhat lower for men (RR men: 0.90, *P* value = 0.084) and significantly lower for women (RR women: 0.83, *P* value = 0.000). Time trends did not change when co-morbidities were accounted for (Additional file 3: Table S2).

**Table 2:** Relative risks of dementia incidence and mortality with and mortality without dementia for the two sexes

Model	Variable	Men				Women			
		RR	*P* value	LCI	UCI	RR	*P* value	LCI	UCI
Dementia incidence	Age	1.13	0.000	1.12	1.13	1.14	0.000	1.14	1.15
	Age*Age	9.99	0.001	9.99	10.00	9.98	0.000	9.97	9.98
	Period								
	2006/2007	1.10	0.006	1.03	1.17	1.10	0.000	1.05	1.15
	2009/2010 (RG)	1		1	1	1		1	1
	Constant	0.03	0.000	0.03	0.03	0.03	0.000	0.03	0.03
	LL	−18,278.2				−34,580.6			
Mortality without dementia diagnosis	Age	1.09	0.000	1.08	1.09	1.11	0.000	1.11	1.12
	Age*Age	10.01	0.000	10.00	10.01	10.01	0.000	10.01	10.01
	Period								
	2006/2007	1.04	0.075	1.00	1.09	1.00	0.935	0.96	1.04
	2009/2010 (RG)	1		1	1	1		1	1
	Constant	0.06	0.000	0.06	0.06	0.03	0.000	0.03	0.03
	LL	−35,303.3				−40,513.3			
Mortality with incident dementia diagnosis	Age	1.05	0.000	1.04	1.06	1.05	0.000	1.04	1.06
	Age*Age	10.01	0.005	10.00	10.02	10.01	0.006	10.00	10.02
	Period								
	2006/2007	0.90	0.084	0.81	1.01	0.83	0.000	0.76	0.91
	2009/2010 (RG)	1		1	1	1		1	1
	Constant	0.37	0.000	0.34	0.41	0.22	0.000	0.21	0.24
	LL	−4,173.1				−6,941.1			

*RR* relative risk, *LCI* 95 % lower confidence interval, *UCI* 95 % upper confidence interval

^+^E-10

### Life years with/without dementia estimated with the illness-death model

Predicted hazard rates from the incidence and mortality models fitted the observed values well (Fig. 2). Using the predicted rates as the input into the illness-death model, we found more years lived healthily and a compression of years with dementia. Among men, trends in incidence and death rates amounted to an increase of 4.8 healthy months (Table 3: LE_w/o dementia 65_ 2006/07: 14.79 years, 2009/10: 15.14 years; difference = 0.35 years) and to a decrease of 1.1 months with dementia (LE _dementia 65:_ 2006/07 0.96 years, 2009/10: 0.87 years, difference = −0.09 years) within 3 years. The increase in healthy life years was weakly significant at *P* = 0.084. Among women, the decrease in months with dementia was highly significant (*P* = 0.000) and stronger than the gain in healthy life months: minus 4.1 months with dementia (LE _dementia 65_ 2006/07: 1.87 years, 2009/10: 1.53 years, difference = −0.34 years) compared with 3.2 healthy months (LE_w/o dementia 65_ 2006/07: 18.14 years, 2009/10: 18.41 years, difference = 0.27 years).

**Table 3:** Life expectancy at age 65 with and without dementia and mean age of new dementia cases, 95 % confidence intervals by sex: 2006/07 in comparison with 2009/10

		Total	Non-dementia	Dementia	Mean age
Males	2006/2007	15.75 (15.58-15.94)	14.79 (14.62–14.96)	0.96 (0.90–1.03)	80.54 (80.27–80.82)
	2009/2010	16.01 (15.85-16.19)	15.14 (14.98–15.32)	0.87 (0.82–0.93)	80.80 (80.53–81.09)
		*P* = 0.224	*P* = 0.084	*P* = 0.180	*P* = 0.446
Females	2006/2007	20.01 (19.86-20.17)	18.14 (18.00–18.29)	1.87 (1.78–1.97)	82.47 (82.29–82.66)
	2009/2010	19.93 (19.77-20.10)	18.41 (18.26–18.56)	1.53 (1.46–1.60)	82.71 (82.52–82.91)
		*P* = 0.706	*P* = 0.148	*P* = 0.000	*P* = 0.310

### Mean age of dementia incidence

**Fig. 3:**
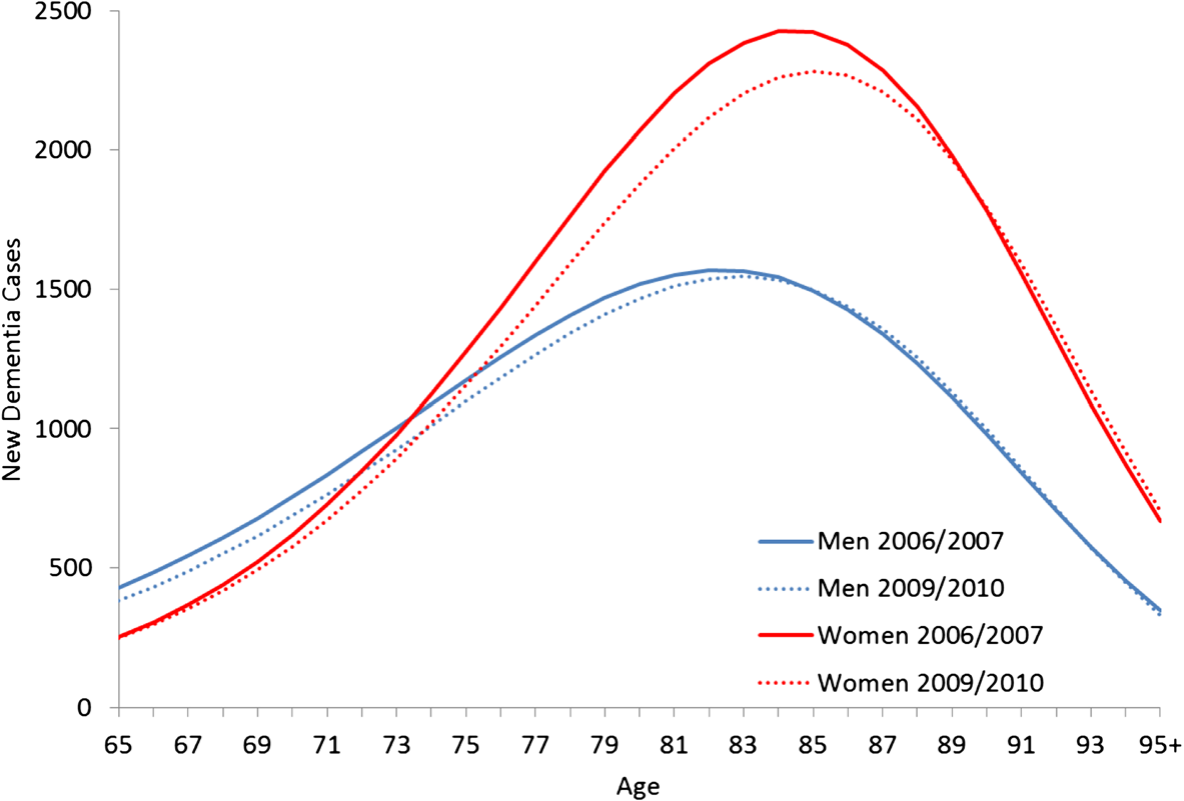
Distribution of new dementia cases in the illness-death model

In the illness-death model, the mean of the distribution of new dementia cases (Fig. 3) increased from 80.54 to 80.80 years among men and 82.47 to 82.71 years among women, albeit this increase was statistically not significant. The variance of the distribution declined from 53.88 to 53.08 for men and increased from 46.85 to 47.99 for women. This was combined with a 3.3 % decrease in the number of new dementia cases among men and 5.0 % among women. Thus, in the latter period, there were less new dementia cases among men and they tended to be compressed into higher ages. Also, among women, there were fewer dementia cases and the mean was shifted to older ages. However, the age distribution became more unequal in terms of variance.

## Discussion

We report a compression of life years with dementia for ages of 65 and above, caused by a significant short-term decrease in the dementia incidence in Germany between 2006/07 and 2009/10. Among both sexes, the compression was intensified by a parallel increase in the death rates of the demented. For women, the significant compression amounted to 1.4 months per year, and men tended to gain almost half a month per year. At the same time, both sexes experienced more healthy life years due to the decreasing incidence. Women gained 1.1 healthy months per year, and men 1.4 months; however, these trends were statistically significant among men only. This declining incidence was associated with an increase in the mean age of dementia incidence.

Our study suggests that declining incidence does not necessarily lead to a concentration of new dementia cases into a smaller age range. Among men, the declining incidence indeed resulted in a concentration of the ages at first diagnosis since the variance of the distribution became smaller; among women; however, the variance increased and the ages at first diagnosis became more variable. Male death rates at the ages studied here are about twice those of females. Thus, men spared from dementia at younger ages die before they suffer from dementia at older ages, whereas comparatively more women survive without dementia and then suffer from dementia at older ages. Owing to the short time period of this study, the observed distributional changes are statistically not significant. Longer time periods are necessary to finally confirm the trends observed here.

### Comparison with results from earlier studies

The decline in dementia incidence reflects results of recent studies [14] (for a review, see [27]). The extent of the yearly 3 % decline is similar to the one reported in the Rotterdam Study [14]. Larson et al. [22] suggest that the main factor contributing to the decline is increasingly higher educational attainment among the elderly, which resulted in increasing cognitive reserve [21]. In the age groups studied here, both sexes profited from the educational expansion after World War I in Germany, and women in particular profited from vocational training from the 1920s to the 1950s [28]. In the HRS [29], the increasing levels of education and wealth among older Americans explained about 40 % of the observed relative decrease in the prevalence of dementia-related cognitive impairment between 1993 and 2002. In the Stockholm Study [15], no changes in the prevalence of dementia or in the death rates of the demented remained once education was accounted for. We do not have information on education in our data and thus are not able to further explore the contribution of education to the declining trend in dementia incidence. We tried to capture possible time trends in vascular risk factors [30] of dementia by including related co-morbidities in our model. Although the effects of the co-morbidities altered the risk of dementia incidence and mortality according to our expectations, they did not affect the estimates of the time trends. Thus, we find no evidence that a change in the distribution of vascular risk factors, as reflected in co-morbidities, contributed to the short-term decline in dementia incidence.

### Trends in dementia incidence

There are at least three potential explanations that may account for the declining dementia incidence we observe. The first explanation assumes that the timing of dementia diagnosis has changed rather than the incidence of the disease. In claims data, the timing of dementia diagnosis is not identical with the exact incidence of the disease. If diagnosis tends to be delayed over time until patients have reached a more severe stage of the disease, then age-specific diagnosis rates would decline. At the same time, those diagnosed would have worse health, leading to an increase in age-specific death rates. As claims data do not contain information about the severity of dementia, we tried to control for a possible delay in the diagnosis by including information about co-morbidity and care need in our models (shown in “Additional file 3: Table S2”). If over the study period dementia diagnosis was delayed to a more severe stage, then patients should suffer from more co-morbidities and should require more care assistance. In the models, the time trend in dementia should thus be attenuated by the inclusion of the two variables. However, this is not what we find, because the time trend remained unchanged. Thus, there is no indication that dementia diagnosis was delayed to a more severe stage. In recent years, public health programs have tried to increase awareness about the disease both in the general population [31] as well as among the medical profession [32]. First generic anti-dementive drugs were introduced in 2012, which should have made treatment less costly but does not fall into our study period.

A second alternative explanation is that the declining incidence reflects an improvement in general health over time. Most recently, it has been suggested that late-life dementia can best be understood in terms of overall health status rather than specific risk factors [19]. A series of studies has shown that physical health measured in terms of functional limitations has improved over time, leading to better functioning in more recent cohorts. At the same time, the prevalence of morbidity has increased (for a review, see Christensen et al. [33]). Our results suggest that the decline in dementia incidence is independent of trends in selected co-morbidities. These trends, however, may in part only show trends in general health and might in part reflect improved medical knowledge and health-service use in elderly people, without changes in underlying conditions [34].

A third explanation is that cognitive reserve had increased over time, which would lead to a decline in incidence rates and an increase in death rates [22]. However, claims data do not contain information about educational or occupational background (or both) of the insurants and thus we were not able to test this hypothesis explicitly. However, we will come back to this potential explanation below.

### Trends in the mortality of patients with dementia

Although only few studies report trends in the mortality of patients with dementia, these tend to report mixed results: increasing trends in health claims data [16, 17] and the HRS [9], a declining trend in the Stockholm Study [15]. However, none of these studies was able to explore sex-specific trends because of the small numbers involved. One explanation for the increasing mortality may be changes in cognitive reserve due to the educational expansion after World War I and the opening of career possibilities after World War II [28]. This would result in a later diagnosis of dementia but also shortened survival thereafter [9, 18]. Trends in educational expansion might also explain why the compression was stronger for women than for men. It has been pointed out that, in the 20th century, German women’s educational levels, labour force participation rates, and access to career opportunities underwent tremendous changes, even more than for men [35]. In addition, European women gained more than men from societal improvements over time, thereby increasing their general cognitive ability more than men [36].

### Trends in risk factors of dementia

Recent studies have highlighted that the reduction of vascular risk factors in mid and late life not only increased LE but also led to a lower risk of late-life dementia [20]. Further progress may thus be derived from public health interventions that lead to changes in lifestyle. Changes in dietary habits [37], such as following the Mediterranean diet more closely [38] and increased physical activity [39], have the highest potential to contribute to a delay in or the prevention of dementia. In addition to single risk factors, it has been shown that overall health status is a major or contributing risk factor of dementia [19] and that a focus on cardiometabolic risk factors may be too limited [40]. Educational levels and wealth have been continuously increasing among today’s elderly [28], and poverty levels are low. Although these trends are expected to continue in the near future, shortened and broken working careers coupled with less generous retirement schemes in the coming generations will eventually lead to higher levels of old-age poverty.

Although our study does not permit any cohort analysis, the positive trends we observe today may be partially due to the favourable mid- and late-life conditions for cohorts born before World War II, to which the study participants belong. This trend may, however, diminish in subsequent generations. The end of World War II may also play an important role in terms of a reduction of people affected by post-traumatic stress disorder (PTSD) throughout the cohorts. With some exceptions, the last German male cohorts actively involved in combat are those born in 1927 who were 17 years old at the end of World War II and were 79 years or older in our study. Cohorts born before 1927 had a high risk of suffering from PTSD due to combat and war imprisonment. Recent research has highlighted the long-term effects of traumatic brain injury and PTSD on dementia later in life [41, 42]. Although little is known about PTSD as a risk factor of dementia, it can be a chronic condition which is related to depression, head injury, or medical co-morbidities, all conditions associated with both PTSD and dementia. An alternative explanation is that PTSD might be related to accelerated brain ageing [43].

In increasingly ethnically diverse older populations, certain ethnic groups may be at a higher risk of dementia, which may slow down improvements in the occurrence of dementia. In Germany, for example, the Turkish foreign population was and still is the largest of the immigrant groups [44]. The majority of the migrants from Turkey arrived as so-called guest workers who were recruited by the Federal Republic of Germany between 1961 and 1973. These migrants are now ageing and in general have similar LE but worse health than their German native counterparts, particularly women. Both the young and the elderly have less education and wealth [45] and higher obesity rates [46]. Rising levels of obesity [47] in combination with metabolic diseases [48] pose another challenge for reducing dementia in the future, particularly in low- and middle-income countries [40]. Public health interventions at the individual and societal level, together with intensified research on developing preventive drugs as well as more effective treatments, will be necessary.

### Strengths and weaknesses of the data

The primary aim of German health claims data is cost calculation and reimbursement rather than documentation of disease. This may lead to various distortions regarding false-positive and false-negative diagnoses. Although we did develop an internal validation procedure to deal with false-positive diagnoses, we were not able to identify false-negative diagnoses. However, a study comparing dementia diagnoses based on Medicare claims data with diagnoses based on [23] found that Medicare claims tend to overestimated the true prevalence of dementia. In a previous study, we showed that the prevalence of dementia compares well with results from international studies [3], which is also true for the incidence in this study. There were no major legal changes or changes in the cost calculation software during the study period, and the increasing knowledge and awareness of physicians and patients cannot explain the decrease in incidence as well as prevalence [3]. Health claims data cover the total population, including people living in nursing homes where the prevalence of dementia is four times higher than for non-residents [49]. Because we use a random sample of the total population, study design or self-selection biases do not exist. The large number of cases allowed us to conduct a detailed analysis of incidence and mortality up through the highest ages. The socio-economic status of the company’s insurants is lower than that of the general population [50], but this is largely among younger people. Changes in the social status composition of the insured population are negligible in the time period studied here.

### Limitations of the illness-death model

Our analysis rests on three assumptions. First, the illness-death model, like all life tables, assumes a stationary population with no changes in age-specific rates [51]; that is, for each of the two periods, LE is calculated under the assumption that the age-specific rates will remain constant until the last member of the cohort has reached the highest age. Because we show changes in dementia incidence and mortality over time, our LEs depict the disease regime in a given year rather than in the future years of a 65-year-old which will be lived healthily or with dementia. If the decline in the dementia incidence continues, then the healthy life years are underestimated, whereas continuous increases in the mortality rate of the demented would cause an overestimation of life years with dementia. The second assumption is that we do not observe the ageing of one specific birth cohort over the whole age range, but rather we use a synthetic cohort constructed from two cross-sections with a short-term follow-up. Thus, our approach is essentially cross-sectional, depicting period rather than cohort LE. The third is that the model does not consider recovery from dementia, which reflects the fact that at present dementia cannot be treated. Mild cognitive impairment is not part of our model. Recent studies show that patients with mild cognitive impairment have a high risk of progressing to dementia, even if they had reverted to normal cognitive functioning [52]. Thus, in our data, we start to include them only once they have received their first dementia diagnosis.

## Conclusions

Using population-based medical claims data for Germany, we found a compression of life years with dementia by a yearly 1.4 months for women and almost half a month for men. The compression was due to declining incidence rates and increasing death rates of the demented. The scale of reduction in the incidence of dementia is comparable to earlier results. In Germany, in the age groups studied here, both sexes profited from the educational expansion after World War I and better living conditions after World War II. In addition, vascular risk factors of dementia have declined and are better controlled. In terms of future numbers of patients with dementia, the increase might be smaller than previously expected. An open question is whether increasing obesity levels and the changing social composition of the elderly might counterbalance positive developments observed in the past.

## Additional files

Additional file 1:
**Method.** Description of the proportional hazard model and the illness-death model. (DOC 401 kb) Additional file 2: Table S1. Descriptive statistics of the exposures in person-years and cases in the two time periods by sex and age group. (DOC 32 kb) Additional file 3: Table S2. Relative risks of dementia incidence, mortality with, and mortality without dementia for the two sexes, controlled for co-morbidity and care level. (DOC 120 kb)

## Abbreviations

AOK: Allgemeine Ortskrankenkassen
HRS: Health and Retirement Survey
ICD-10: International Classification of Disease 10th Revision
LE: Life expectancy
LTC: long-term care
PTSD: Post-traumatic stress disorder
PYE: Person-years of exposure
RR: Relative risk
WIdO: Wissenschaftliches Institut der Ortskrankenkassen
