# Supplementary material for: Compression or expansion of dementia in Germany? An observational study of short-term trends in incidence and death rates of dementia between 2006/07 and 2009/10 based on German health insurance data
Source: Alzheimers Res Ther. 2015 Nov 5;7:66. doi: 10.1186/s13195-015-0146-x (PMC4634148; doi:10.1186/s13195-015-0146-x)
Supplement: Additional file 1: — Method. Description of the proportional hazard model and the illness-death model. (DOC 401 kb) [file 13195_2015_146_MOESM1_ESM.doc]

Method

Estimation of the relative risks of period 2009/10 as compared to 2006/7

We used an exponential survival model with a constant baseline hazard. That means that the instantaneous potential for dementia or death remains constant during the study period which in our case is 2006/7 and 2009/10. In general the equation for the calculation of the hazard rate in the exponential model is:


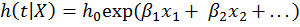


The hazard rate
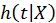
 is the dependent variable. It describes the duration of time from an initial state to reaching a goal state. The baseline hazard
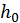
 remains constant during the time scale which is time in months since 2006 (2009). For the estimation of the regression coefficients
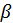
 the maximum likelihood method was used. The model included age at incident dementia /death as a 2^nd^ degree polynomial , and an indicator variable for the two time periods where the period 2006/7 takes the value one and zero otherwise. In a sensitivity analysis we included time-dependent indicator variables for comorbidities which take the value one from the first time comorbidity was diagnosed and zero otherwise. We used the procedure “streg” in STATA 12.1 for the estimation of the hazard models.

Illness-death model

To calculate the illness-death model we first estimated the age-specific hazard rates of the three transitions (non-dement to dement; non-dement to death; dement to death) by using separate exponential survival models (men 2006/07; women 2006/07; men 2009/10; women 2009/10). In these models we only included the 2^nd^ degree age-polynomials as covariates. We used the postestimation command “predict” for the prediction of the hazard rates. The fit of the models is displayed in Figure 1.

Then we used the predicted age-specific hazard rates (
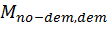
;
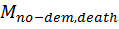
;
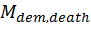
) to calculate separate illness-death models for men and women in the years 2006/07 and 2009/10.

1. The total probability of leaving the non-demented state in a one year age interval was calculated from the transition from non-demented to death and the transition from non-demented to dement.


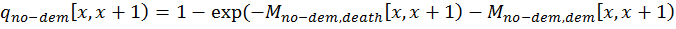


1. The probability of transit from the non-demented state to the demented state was calculated by:


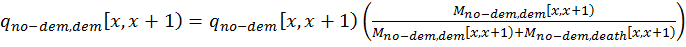


1. The probability of transit from the non-demented state to death was estimated similarly by using:


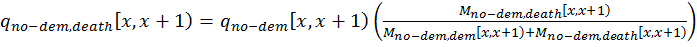


1. The probability to transit from the demented state to death was calculated by:


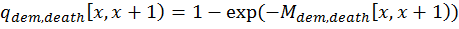


1. The survival probability
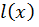
at exact age x was calculated by using a radix (starting number of life table entries) of 100 000 persons at age 65. This total of 100 000 people was divided into non-demented and demented by taking the prevalence rates of dementia at age 65 in the first quarter of 2006 (men 1.66 and women 1.40 cases per 100 persons) and accordingly in the first quarter of 2009 (men 1.59 and women 1.13 cases per 100 persons). According to this we assumed for men
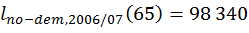
;
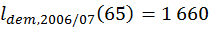
;
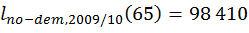
;
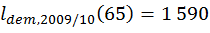
 and for women
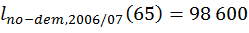
;
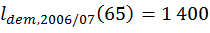
;
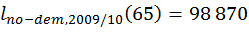
;
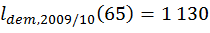
. The probability of surviving in the non-demented and the demented state at age x+1 was calculated by:


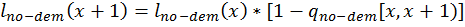


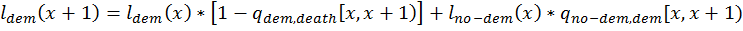


Thereby the sum of
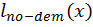
 and
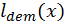
 is the total probability of surviving at age x (
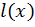
).

1. The number of person years lived between the ages of x and x+1 in the states non-demented and demented was calculated by:


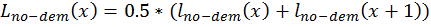


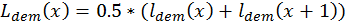
.

1. Our life tables were closed at age 95. We assumed that


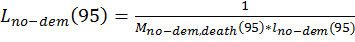


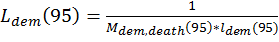
 .

1. The total number of person years lived
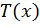
 in the non-demented and demented states is:


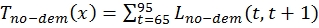


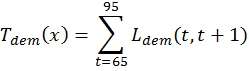


and life expectancy at age x is


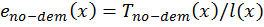


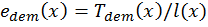


1. The number of new dementia cases
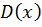
 at age x is


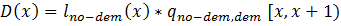
.
