# Supplementary material for: Compression or expansion of dementia in Germany? An observational study of short-term trends in incidence and death rates of dementia between 2006/07 and 2009/10 based on German health insurance data
Source: Alzheimers Res Ther. 2015 Nov 5;7:66. doi: 10.1186/s13195-015-0146-x (PMC4634148; doi:10.1186/s13195-015-0146-x)
Supplement: Additional file 2: Table S1. — Descriptive statistics of the exposures in person-years and cases in the two time periods by sex and age group. (DOC 32 kb) [file 13195_2015_146_MOESM2_ESM.doc]

Additional file 2: Table S1: Descriptive statistics of the exposures in person-years and cases in the two time periods by sex and age group
