# Supplementary material for: Compression or expansion of dementia in Germany? An observational study of short-term trends in incidence and death rates of dementia between 2006/07 and 2009/10 based on German health insurance data
Source: Alzheimers Res Ther. 2015 Nov 5;7:66. doi: 10.1186/s13195-015-0146-x (PMC4634148; doi:10.1186/s13195-015-0146-x)
Supplement: Additional file 3: Table S2. — Relative risks of dementia incidence, mortality with, and mortality without dementia for the two sexes, controlled for co-morbidity and care level. (DOC 120 kb) [file 13195_2015_146_MOESM3_ESM.doc]

Additional file 3: Table S2: Relative risks of dementia incidence, mortality with, and mortality without dementia for the two sexes, controlled for comorbidity and care level

| Model | Variable | Men | | | | Women | | | |
| --- | --- | --- | --- | --- | --- | --- | --- | --- | --- |
|  |  | RR | p-value | LCI | UCI | RR | p-value | LCI | UCI |
| Dementia Incidence | Age | 1.07 | 0.000 | 1.07 | 1.08 | 1.08 | 0.000 | 1.08 | 1.09 |
|  | Age^2+^ | 9.98 | 0.000 | 9.97 | 9.98 | 9.97 | 0.000 | 9.96 | 9.97 |
|  | *Period* |  |  |  |  |  |  |  |  |
|  | 2006/2007 | 1.09 | 0.007 | 1.02 | 1.17 | 1.10 | 0.000 | 1.05 | 1.15 |
|  | 2009/2010 (RG) | 1 |  | 1 | 1 | 1 |  | 1 | 1 |
|  | *Care level* |  |  |  |  |  |  |  |  |
|  | 0 (RG) | 1 |  | 1 | 1 | 1 |  | 1 | 1 |
|  | 1 | 5.35 | 0.000 | 4.91 | 5.84 | 4.32 | 0.000 | 4.07 | 4.59 |
|  | 2 | 8.13 | 0.000 | 7.39 | 8.93 | 6.12 | 0.000 | 5.72 | 6.55 |
|  | 3 | 10.26 | 0.000 | 8.71 | 12.08 | 7.50 | 0.000 | 6.70 | 8.39 |
|  | *Co-morbidities* |  |  |  |  |  |  |  |  |
|  | Hypertension | 1.06 | 0.207 | 0.97 | 1.17 | 1.01 | 0.690 | 0.94 | 1.09 |
|  | Diabetes | 1.12 | 0.001 | 1.04 | 1.20 | 1.14 | 0.000 | 1.09 | 1.20 |
|  | Ischemic heart disease | 1.05 | 0.190 | 0.98 | 1.13 | 1.03 | 0.285 | 0.98 | 1.08 |
|  | Cerebrovascular disease | 1.90 | 0.000 | 1.77 | 2.04 | 1.80 | 0.000 | 1.72 | 1.89 |
|  | Cholesterol | 0.89 | 0.004 | 0.82 | 0.96 | 0.93 | 0.006 | 0.88 | 0.98 |
|  | Atrial fibrillation | 1.39 | 0.000 | 1.29 | 1.50 | 1.32 | 0.000 | 1.25 | 1.39 |
|  | *Constant* | 0.01 | 0.000 | 0.01 | 0.01 | 0.01 | 0.000 | 0.01 | 0.01 |
|  | LL | -16,487.1 |  |  |  | -31,821.2 |  |  |  |
| Mortality without dementia diagnosis | Age | 1.03 | 0.000 | 1.02 | 1.03 | 1.04 | 0.000 | 1.04 | 1.04 |
|  | Age^2+^ | 9.99 | 0.006 | 9.99 | 10.00 | 10.00 | 0.029 | 9.99 | 10.00 |
|  | *Period* |  |  |  |  |  |  |  |  |
|  | 2006/2007 | 1.04 | 0.052 | 1.00 | 1.09 | 1.02 | 0.304 | 0.98 | 1.07 |
|  | 2009/2010 (RG) | 1 |  | 1 | 1 | 1 |  | 1 | 1 |
|  | *Care level* |  |  |  |  |  |  |  |  |
|  | 0 (RG) | 1 |  | 1 | 1 | 1 |  | 1 | 1 |
|  | 1 | 4.58 | 0.000 | 4.29 | 4.88 | 4.17 | 0.000 | 3.93 | 4.43 |
|  | 2 | 11.29 | 0.000 | 10.61 | 12.02 | 10.90 | 0.000 | 10.27 | 11.57 |
|  | 3 | 24.81 | 0.000 | 22.74 | 27.07 | 24.54 | 0.000 | 22.73 | 26.50 |
|  | *Co-morbidities* |  |  |  |  |  |  |  |  |
|  | Hypertension | 1.02 | 0.455 | 0.96 | 1.09 | 1.10 | 0.008 | 1.02 | 1.17 |
|  | Diabetes | 1.20 | 0.000 | 1.15 | 1.26 | 1.20 | 0.000 | 1.15 | 1.25 |
|  | Ischemic heart disease | 1.33 | 0.000 | 1.27 | 1.40 | 1.20 | 0.000 | 1.15 | 1.26 |
|  | Cerebrovascular disease | 1.04 | 0.111 | 0.99 | 1.09 | 1.15 | 0.000 | 1.10 | 1.21 |
|  | Cholesterol | 0.89 | 0.000 | 0.85 | 0.94 | 0.88 | 0.000 | 0.84 | 0.93 |
|  | Atrial fibrillation | 2.05 | 0.000 | 1.95 | 2.16 | 2.15 | 0.000 | 2.05 | 2.25 |
|  | *Constant* | 0.02 | 0.000 | 0.02 | 0.02 | 0.01 | 0.000 | 0.01 | 0.01 |
|  | LL | -30,577.2 |  |  |  | -34,769.2 |  |  |  |
|  |  |  |  |  |  |  |  |  |  |
|  |  |  |  |  |  |  |  |  |  |
| Mortality with dementia diagnosis | Age | 1.04 | 0.000 | 1.03 | 1.05 | 1.03 | 0.000 | 1.02 | 1.04 |
|  | Age^2+^ | 10.00 | 0.357 | 10.00 | 10.01 | 10.01 | 0.085 | 10.00 | 10.02 |
|  | *Period* |  |  |  |  |  |  |  |  |
|  | 2006/2007 | 0.96 | 0.447 | 0.85 | 1.07 | 0.88 | 0.006 | 0.80 | 0.96 |
|  | 2009/2010 (RG) | 1 |  | 1 | 1 | 1 |  | 1 | 1 |
|  | *Care level* |  |  |  |  |  |  |  |  |
|  | 0 (RG) | 1 |  | 1 | 1 | 1 |  | 1 | 1 |
|  | 1 | 1.73 | 0.000 | 1.46 | 2.05 | 1.58 | 0.000 | 1.36 | 1.82 |
|  | 2 | 3.39 | 0.000 | 2.91 | 3.96 | 3.31 | 0.000 | 2.89 | 3.79 |
|  | 3 | 6.25 | 0.000 | 5.17 | 7.55 | 5.71 | 0.000 | 4.87 | 6.70 |
|  | *Co-morbidities* |  |  |  |  |  |  |  |  |
|  | Hypertension | 3.74 | 0.000 | 3.02 | 4.65 | 3.10 | 0.000 | 2.60 | 3.70 |
|  | Diabetes | 1.41 | 0.000 | 1.18 | 1.68 | 1.23 | 0.007 | 1.06 | 1.42 |
|  | Ischemic heart disease | 1.16 | 0.118 | 0.96 | 1.41 | 1.28 | 0.002 | 1.09 | 1.50 |
|  | Cerebrovascular disease | 0.98 | 0.790 | 0.82 | 1.16 | 1.06 | 0.473 | 0.91 | 1.23 |
|  | Cholesterol | 1.25 | 0.027 | 1.03 | 1.52 | 0.93 | 0.451 | 0.78 | 1.12 |
|  | Atrial fibrillation | 1.49 | 0.000 | 1.25 | 1.78 | 1.86 | 0.000 | 1.60 | 2.16 |
|  | *Constant* | 0.12 | 0.000 | 0.11 | 0.14 | 0.08 | 0.000 | 0.07 | 0.09 |
|  | LL | -3,571.5 |  |  |  | -6,139.5 |  |  |  |
| RR: relative risk; LCI: 95% Lower confidence interval; UCI: 95% Upper confidence interval; ^+^: E-10 | | | | | | | | |  |
